# Lattice Radiation Therapy as a Novel Approach for Renal Cell Carcinoma: A Case Series

**DOI:** 10.7759/cureus.107174

**Published:** 2026-04-16

**Authors:** Spoorthi Vallamkonda, Jennifer S Chiang, Mark K Buyyounouski, Michael S Binkley, Hilary P Bagshaw

**Affiliations:** 1 Department of Radiation Oncology, Stanford University School of Medicine, Stanford, USA

**Keywords:** kidney cancer, lattice radiation therapy, renal cell carcinoma, spatially fractionated radiation therapy, tumor control

## Abstract

Spatially fractionated radiation therapy (SFRT) is a treatment modality initially designed for large, deep-seated tumors. Lattice radiation therapy (LRT), a form of SFRT, delivers high and low doses of radiation (RT) in “peaks” and “valleys,” utilizing a three-dimensional pattern while protecting the surrounding organs at risk. We present a case series of two patients with large renal tumors, which were treated with LRT as follows: a 30-year-old male with metastatic renal cell carcinoma (RCC) and a 72-year-old male with RCC and several other comorbidities who completed LRT with minimal toxicity. Both patients received 6670 cGy in 5 fractions delivered twice weekly. These cases support the potential role of LRT in tumor control and symptom palliation. Further research is warranted to delineate the long-term efficacy and toxicity profile of LRT.

## Introduction

Each year, more than 81,000 people in the United States are diagnosed with kidney cancer [1]. Renal cell carcinoma (RCC) accounts for up to 85% of all kidney cancers in adults and is twice as common in men as in women [2]. While most individuals diagnosed are treated for localized disease, 25-50% eventually develop metastatic disease, limiting certain therapies [3]. Bulky tumors also present a therapeutic challenge, especially when surgical resection is not possible. While RT is a noninvasive alternative, especially for patients with significant comorbidities or systemic therapy resistance, dose escalation is often required to optimize local control of these radioresistant tumors. Stereotactic body radiation therapy (SBRT) is one ablative option for these patients, offering improved symptom palliation across cancer types and extended survival in oligometastatic populations [3]. However, homogenous dose delivery to these types of tumors may not be as effective due to tumor heterogeneity and limitations posed by surrounding organs at risk (OAR) [4-6].

The large radiation field required to encompass both the tumor's size and volume calls for differentiated RT delivery [4,5]. Spatially fractionated radiation therapy (SFRT) is an RT technique in which specialized beam collimation creates high-dose peaks distributed throughout a target volume, with intervening low-dose valleys, and may enable safe dose escalation for large tumors [7]. Biologically, SFRT elicits cytotoxic effects and alters gene expression through bystander mechanisms [7,8].

The more commonly used form of SFRT, grid RT (GRT), is planned using a 2D technique and may be delivered using either precast blocks or multileaf collimators (MLCs) [9]. A precast block contains a fixed pattern of openings that allows RT to pass through only selected regions of the tumor while blocking others. Alternatively, MLCs are computer-controlled metal leaves that move in and out of the beam to shape a similar grid-like pattern without the need for a physical block. In this way, GRT delivers RT via a limited number of beam directions and produces a 2D pattern of alternating high- and low-dose regions across the tumor cross-section. GRT has been evaluated for large tumors of various histologies in both definitive and palliative settings and is associated with excellent local control and low toxicity [5,10-17].

In contrast, lattice radiation therapy (LRT) is a modern 3D form of SFRT in which multiple converging photon beams are used to generate discrete high-dose regions, or vertices, positioned within the tumor and separated by specified distances [14,18]. Rather than generating a 2D grid across a single plane, LRT produces a 3D peak-and-valley dose distribution throughout the tumor volume. This approach improves dose conformality and may be more beneficial for large or deep-seated tumors surrounded by OARs, as it allows spatially fractionated high-dose delivery while better limiting dose to surrounding normal tissues [14,18]. Thus, LRT may be considered a more conformal, volumetric evolution of GRT. In this paper, we report on two cases of large renal tumors that were treated with LRT. Both patients tolerated LRT well with no residual treatment toxicities upon follow-up.

## Case presentation

Case 1

**Figure 1:**
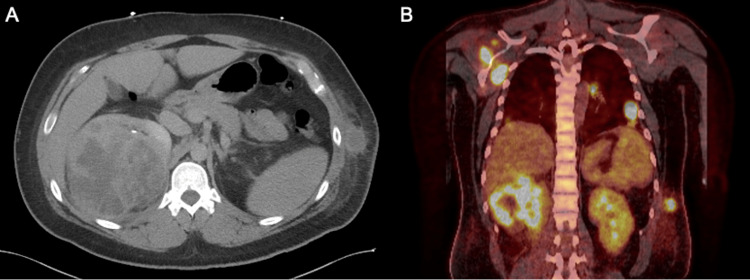
Case 1 - imaging of the right renal mass at initial presentation. (A) Axial computed tomography of the chest, abdomen, and pelvis demonstrates a heterogeneous, complex mass at the upper pole of the right kidney. (B) Positron emission tomography/computed tomography shows intense hypermetabolic activity of the right renal mass, with additional FDG-avid lesions in the lungs, scapula, and left lateral chest wall. FDG: fluorodeoxyglucose

A 30-year-old man with no relevant past medical history presented to his primary care physician with severe anemia, intermittent fevers, a 30-pound weight loss, and dark brown urine for several months. Computed tomography (CT) of the chest, abdomen, and pelvis revealed a right renal mass measuring greater than 11 cm, mediastinal lymphadenopathy, and bilateral pulmonary, left abdominal wall, and left paraspinal nodules (Figure 1, panel A). Biopsy of the left abdominal wall nodule confirmed metastatic clear cell renal cell carcinoma with positive von Hippel-Lindau (VHL) somatic mutation. PET/CT revealed additional metastases in the liver and bone (Figure 1, panel B).

The patient initiated nivolumab (q14 days) and cabozantinib 40 mg daily for one year with subsequent improvement in poor appetite, tachycardia, and night sweats. Interval imaging demonstrated a partial response overall, with residual increased metabolic activity of the right renal mass. However, his immunotherapy treatment course was intermittently held due to complications, including pharyngitis, dermatitis, and perirectal abscess. Approximately one year later, MRI demonstrated enlargement of the right renal mass, now measuring 15.5 cm with increasing solid nodular satellite lesions within the right kidney and direct invasion into the right psoas and quadratus. Upon multidisciplinary review, the decision was made to delay surgery and transition to lenvatinib 18 mg and everolimus 5 mg daily.

As the patient was experiencing significant right back pain uncontrolled by oxycodone, he was then referred to the radiation oncology department for consideration of radiation therapy (RT). Due to the large size of the tumor, it was discussed with the patient that higher doses may offer improved symptom palliation and local control, as has been shown across cancer types. However, surrounding organs at risk may limit the potential for dose escalation. Thus, the patient was offered and elected to proceed with spatially fractionated radiation therapy (SFRT) in 5 fractions (delivered twice weekly), using the lattice radiation technique (LRT), to the right kidney mass. Lenvatinib and everolimus were held one week prior to radiation treatment, with instructions to resume one week after treatment.

**Figure 2:**
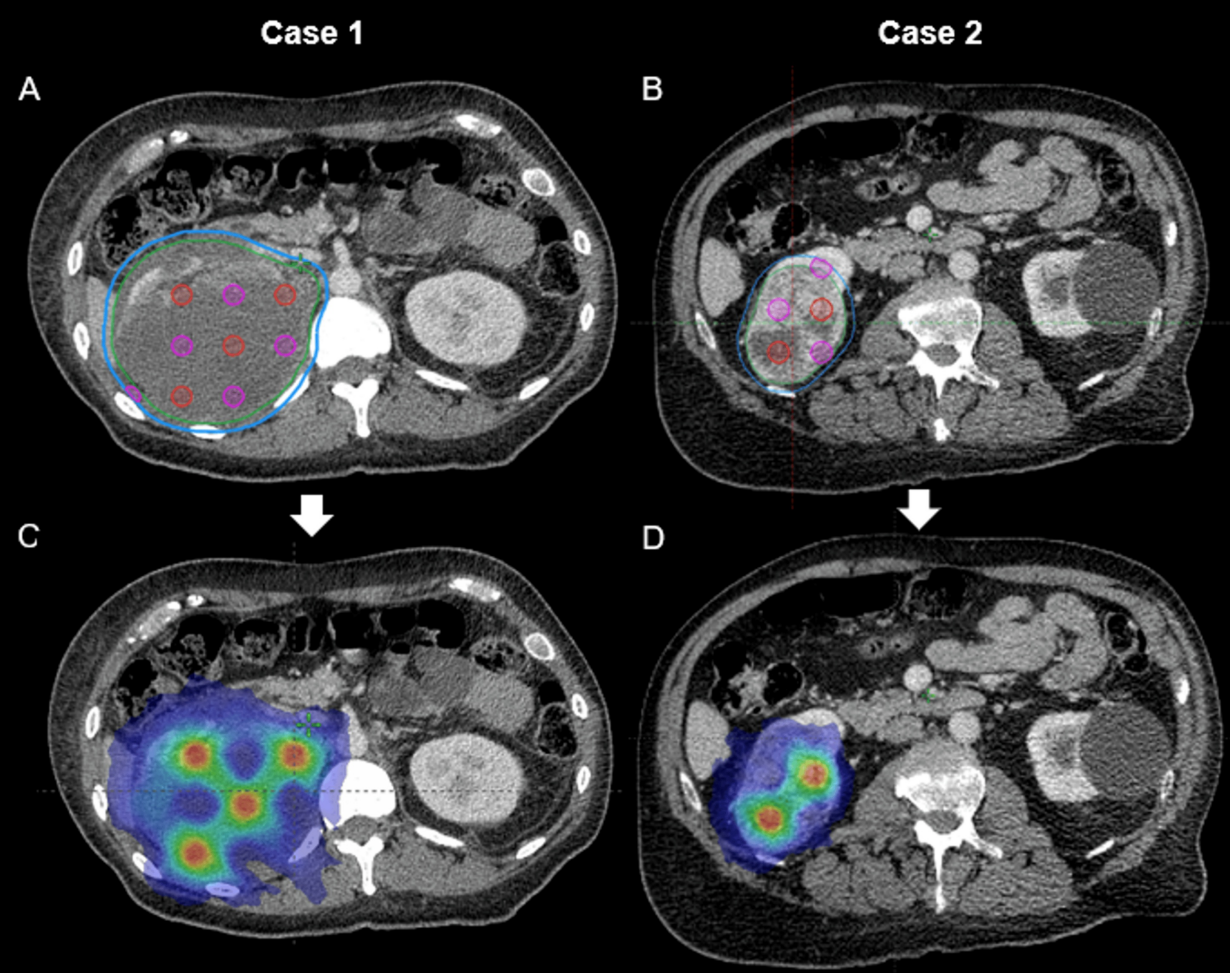
Case 1 and Case 2 - lattice radiotherapy plans. (A and B) Axial computed tomography slices of the targeted right renal mass, with green gross tumor volume prescribed 2000 cGy (GTV_2000) and blue planning tumor volume prescribed 2000 cGy (PTV_2000). Red spheres represent the 1.5-cm-diameter targets delineated as planning target volumes, boosted to 6670 cGy (PTV_6670). Pink spheres represent the avoidance structures delineated as PTV_Avoid. (C and D) Dose distribution after volumetric modulated arc therapy planning for the target (2000 cGy in blue, 6670 cGy in red).

The patient underwent 4D CT simulation in the supine position, with instructions to limit oral intake to medications and water at least 2 h prior to the simulation and treatments. Minimal movement of the renal mass was noted, and the 3D CT with contrast, fused with the patient’s MRI, was used for RT planning, with the technique developed by Duriseti et al. [14]. The right kidney mass was delineated as the gross tumor volume receiving at least 2000 cGy (GTV_2000), with a 5 mm direct expansion to the planning target volume (PTV_2000; Figure 2, panel A). GTV_2000 measured 1,024 cm^3^ in volume. A simultaneously integrated boost of 6670 cGy was prescribed to the peripheries of 1.5 cm diameter spheres (PTV_6670) arranged on a 3x3x3 cm grid overlaid on the largest cross dimension within the GTV_2000. Each PTV_6670 sphere alternated with 1.5 cm diameter avoidance spheres (PTV_Avoid), and a distance of 3 cm was maintained between the center of each PTV_6670 and adjacent PTV_Avoid sphere.

**Figure 3:**
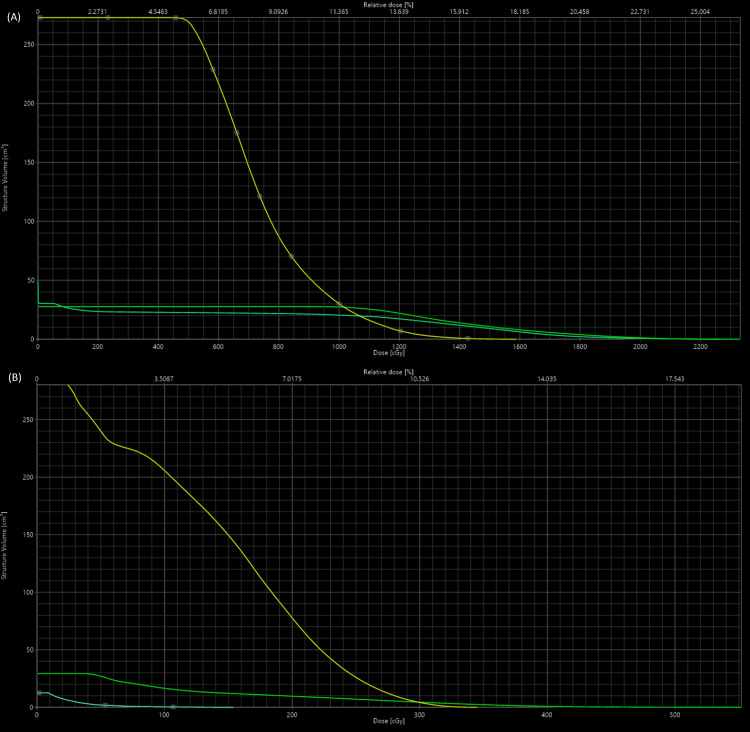
Case 1 and Case 2 - dose-volume histograms for the RT plans. Dose-volume histograms for the RT plans of (A) Case 1 and (B) Case 2, demonstrating dose distributions to selected organs at risk, with the duodenum shown in green, spinal cord in cyan, and kidney contralateral to the target in yellow.

Figure 2, panel C, demonstrates an axial view of the volumetric modulated arc therapy (VMAT) plan. The plan achieved 100% coverage of PTV_2000 with 2000 cGy and was normalized to 95% coverage of PTV_6670 with 6670 cGy. Key dose constraints, similar to those typical of 5-fraction renal stereotactic body radiation therapy (SBRT) for RCC, were achieved for the spinal cord (Dmax <28 Gy; V22.5 <0.25 cc), duodenum (Dmax <26 Gy, V18 <5 cc), and bilateral renal cortices (V17.5 <200 cc). Figure 3, panel A, demonstrates the dose-volume histogram of the RT plan. During RT, lenvatinib and everolimus were held to avoid potential compounding toxicities. The patient tolerated the treatment well, with mild reported toxicities of fatigue and nausea controlled with ondansetron as needed.

Notably, one week after completion of RT, the patient was hospitalized after presenting with palpitations and diagnosed with sepsis (Common Terminology Criteria for Adverse Events {CTCAE} v5.0 grade 4) secondary to Salmonella bacteremia, thought to originate from a renal fluid collection. The MR abdomen during hospitalization demonstrated a decreased size of the right kidney mass, measuring 11 cm. He was treated with pressors, IV antibiotics, and drain placement prior to discharge, two weeks after admission. Due to abnormal bone alkaline phosphatase upon admission and previous intolerable side effects of weight loss and decreased appetite, everolimus was not restarted, and the patient transitioned to lenvatinib and pembrolizumab one week after discharge. At his six-week follow-up visit, he reported improved pain, no longer required oxycodone, and controlled with acetaminophen. CT imaging two months post-RT demonstrated a stable size of the treated mass.

Case 2

**Figure 4:**
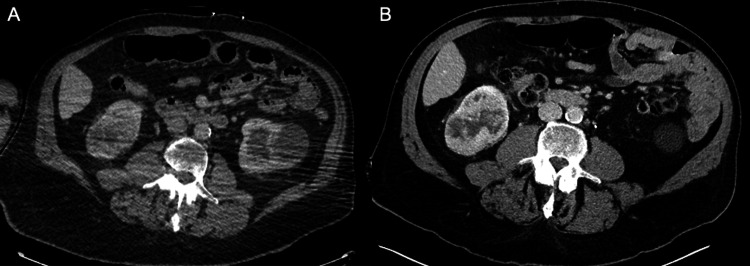
Case 2 - imaging of the right renal mass prior to radiation therapy. (A) Axial computed tomography (CT) of the chest, abdomen, and pelvis demonstrates a mixed solid and cystic mass at the lower pole of the right kidney. (B) Follow-up CT five months later shows interval enlargement of the mass.

A 72-year-old man with Parkinson's disease, dementia, and orthostatic hypotension presented to the emergency department after an unwitnessed fall. CT of the chest, abdomen, and pelvis revealed a solid and cystic right renal lower pole mass measuring 6.2x6.4x4.8 cm with an adjacent similar appearing interpolar mass measuring 3.0x2.4x2.2 cm (Figure 4, panel A). There was no evidence of metastatic disease. Upon establishment of care with the urology department, the likelihood of malignancy was discussed; biopsy was not recommended, as it was unlikely to change management. Six months later, CT of the abdomen and pelvis revealed enlargement of the lower pole mass, measuring up to 8.2 cm (Figure 4, panel B).

Due to his age, comorbidities, and poor performance status, the patient was not a surgical candidate and thus referred to the radiation oncology department for consideration of RT, with multidisciplinary consensus. Given the recent growth of the tumor, SFRT was offered to maximize local control. The patient elected to proceed and underwent a 3D CT simulation with contrast in the supine position, following standardized fasting instructions. Target volumes were delineated as demonstrated in Figure 2, panel B. The gross disease of the right kidney was contoured as the GTV_2000, measuring 222 cm^3^. A 5 mm expansion upon the GTV_2000 was used to delineate the PTV_2000.

The patient was treated with five weekly fractions of SFRT to 2000 cGy with a simultaneously integrated boost to 6670 cGy (Figure 2, panel D). The previously described treatment optimization parameters in Case 1 were similarly achieved in this patient’s RT plan, the DVH of which is represented in Figure 3, panel B. The patient tolerated the treatment well with short-term side effects, including mild nausea and fatigue, for which conservative management was sufficient. Three months after completion of RT, the patient reported no residual toxicities of the treatment. The patient was recommended to undergo follow-up imaging three months after completion of radiation, the completion of which is pending.

## Discussion

Mortality rates of kidney cancer have decreased by approximately 2% every year from 2016 to 2020, highlighting the impact of new treatment modalities and technologies [19]. However, patients with bulky and/or metastatic disease continue to have significant morbidity and mortality due to more limited treatment options [14]. SFRT delivers precise RT to tumors using vertices of high ablative doses while protecting nearby organs at risk (OARs).

To our knowledge, the presented work includes the largest tumor successfully treated with this regimen of LRT (2000 cGy in 5 fractions with a simultaneously integrated boost to 6670 cGy) to date. In the first case presented, the patient received LRT to a total dose of 6670 cGy in 5 fractions and experienced both radiographic response and pain palliation. The maximal tumoral dimension was 15.5 cm prior to treatment and decreased to 11 cm at two-month follow-up. He was also able to de-escalate his pain management from requiring oxycodone to using Tylenol as needed. In the second case presented, the patient tolerated a similar regimen of LRT, with only anticipated mild acute side effects, including fatigue. Although this patient did not require pre-medication, pre-medication may be administered on a case-by-case basis when there is concern for tolerability, baseline symptom burden, or anticipated discomfort with treatment.

Overall, these cases support LRT as an effective treatment modality for tumor control and symptomatic palliation. While systemic therapy (lenvatinib and everolimus) may have influenced clinical outcomes of the first case, additional studies are needed to further characterize the relationship between chemotherapy and LRT; in one recent retrospective study, concurrent systemic therapy was not found to be significantly associated with improved treatment response (clinical or radiographic) [10]. As shown in the two cases, this method has proven successful for dose escalation and heterogeneous RT delivery, demonstrating its value in the inoperable setting for radioresistant cancers like RCC.

Additionally, these cases illustrate how LRT may be safely delivered in different clinical contexts. In the first, the patient had significant difficulty with tolerating previous systemic therapies, requiring multiple treatment breaks, yet was able to complete LRT with minimal toxicity and achieve tumor control. Importantly, although the patient was hospitalized one week after completion of LRT for sepsis in the setting of tumor necrosis, the etiology was considered likely multifactorial, as a more gradual necrosis timeline would generally be expected in this slow-growing tumor if it were directly attributable to RT alone. In the second case, an older patient who was initially put on surveillance and later recommended for definitive management was not an ideal surgical candidate due to his multiple comorbidities. He was able to undergo and tolerate LRT well. While limited to two cases, these experiences suggest that LRT can be feasible in select patients with otherwise challenging treatment scenarios, with the potential advantage of managing bulky tumors in a less invasive manner and with minimal toxicity, thereby supporting quality of life.

Recent clinical studies provide additional evidence supporting the role of LRT in managing bulky, treatment-resistant tumors. In 2022, a multicenter prospective study, LATTICE-01, investigated the use of the lattice technique in patients with lesions >5 cm and stage IV cancer [5]. All patients were unsuitable for surgical resection or ablative stereotactic irradiation. The study included a total of 30 patients, with 10% having kidney cancer. A median dose of 1500 cGy in 1 fraction (range: 1000 cGy in 1 fraction - 2700 cGy in 3 fractions) was delivered to the vertices within the GTV. At a median follow-up of 10.75 months, rates of symptomatic and clinical response were 100% and 89%, respectively, and 23% of patients achieved complete remission.

A phase one trial titled LITE SABR M1 investigated 22 patients with tumors at least 4.5 cm in diameter, treated with LRT to 2000 cGy in 5 fractions, every other day, with a simultaneous integrated boost of 6670 cGy [20]. Toxicity and quality of life were measured at 14 and 90 days after treatment. The Patient-Reported Outcomes Measurement Information System (PROMIS) was used to assess anxiety, depression, pain interference, physical global health, and physical function at the following four time points: baseline and 14-, 30-, and 90-day follow-ups. At baseline, all individuals exhibited higher levels of these parameters compared to healthy individuals, and throughout follow-up, all quality of life (QoL) measures improved. No grade 3 toxicities, aside from one grade 4 urosepsis following treatment of a retroperitoneal tumor, were observed within 90 days.

Most recently, Wang et al. conducted a retrospective study of 80 patients with locally advanced or metastatic bulky tumors (≥6 cm in diameter) treated with grid RT (GRT), using either grid block RT (62%) or grid intensity-modulated radiation therapy (IMRT) (38%) [10]. A wide range of disease and treatment sites were included, and treatment intent was split evenly between palliative and definitive care. At a median follow-up of eight months, response data from 63 patients showed a 71% average reduction in tumor size, 83% imaging response rate, 79% clinical response rate, and 70% freedom from locoregional recurrence rate. The most common acute toxicities were dermatitis and oral mucositis, while the most common late toxicities were fatigue, pain, and dry mouth. Two patients experienced fatal carotid blowouts within segments of the carotid encompassed by high-dose isodose lines, supporting caution when considering GRT for patients with comorbidities independently associated with vascular events.

## Conclusions

The two cases presented demonstrated clinical benefits of LRT in terms of tolerability, local control, and palliation. While limited by short-term follow-up, LRT showed promising results in one patient with a bulky tumor resistant to previous systemic therapy (which had also been causing intolerable side effects) and another with significant predisposing comorbidities limiting alternative therapies. In these two cases, radiation therapy was recommended due to its effectiveness in preventing symptomatic progression and in locally controlling primary renal tumors. Due to their large tumor sizes, neither patient was amenable to SBRT due to the large tumor size. LRT was a suitable option due to its effectiveness in limiting toxicity, sparing OARs, and preventing symptomatic progression. This report adds to the growing body of data on potential candidates for this treatment modality. Additional studies, including larger patient cohorts with longer follow-up, are needed to further define the side effect profile and efficacy of LRT.
